# The Relevant Role of Navigated Tractography in Speech Eloquent Area Glioma Surgery: Single Center Experience

**DOI:** 10.3390/brainsci11111436

**Published:** 2021-10-28

**Authors:** Luca Francesco Salvati, Raffaele De Marco, Giuseppe Palmieri, Massimiliano Minardi, Armando Massara, Alessandro Pesaresi, Bernarda Cagetti, Antonio Melcarne, Diego Garbossa

**Affiliations:** 1 Department of Neurosurgery, Santa Corona Hospital, Asl2 Liguria, 17027 Pietra Ligure, Italy; b.cagetti@asl2.liguria.it; 2 Neurosurgery Unit, AOC Città della Salute e della Scienza, Department of Neuroscience “Rita Levi Montalcini”, University of Turin, 10126 Turin, Italy; Raffaele.demarco@unito.it (R.D.M.); giuseppepalm90@alice.it (G.P.); massimominardi91@gmail.com (M.M.); armymax@hotmail.it (A.M.); alessandro.pesaresi@unito.it (A.P.); anmelcarne@gmail.com (A.M.); diego.garbossa@unito.it (D.G.)

**Keywords:** tractography, image-guided neurosurgery, glioma, language pathway

## Abstract

Background: Gliomas are among the most challenging pathologies for neurosurgeons due to their infiltrative and recurrent nature in functionally relevant regions. Current knowledge confirms that gross total resection highly influence survival in patient with glioma. However, surgery performed in eloquent brain area, could seriously compromise the quality of life in patient with reduced life expectancy even more if it concerns the language function. Methods: 18 right-handed patients with perisylvian gliomas on the left hemisphere were prospectively analyzed over a period of 12 months. Standardized preoperative Diffusion-Tensor-Imaging based tractography of the five main language Tracts (Arcuate Fasciculus, Frontal Aslant Tract, Inferior Fronto-Occipital Fasciculus, Inferior Longitudinal Fasciculus, Uncinate Fasciculus) was navigated during the surgical procedure. Using a validated method, correlations were made between the pre-operative fascicles and their possible infiltration and surgical damage. The language status was assessed using the Aachen Aphasia Test. Results: In all nine patients who developed a permanent disorder there was pre-operative involvement of at least one fascicle and resection of at least one of these. In this way, areas of high risk of permanent language damage have emerged as a result of surgical injury: the temporoparietal junction, the middle portion of the FAT and the temporal stem. Conclusions: Navigated tractography has proven to be a user-friendly tool that can assess perioperative risk, guide surgical resection, and help the neurosurgeon to find that balance between tumor resection and function preservation.

## 1. Introduction

Considering the demographic characteristics and overall survival of patients affected by low-grade glioma (LGG), the need to guarantee a good quality of life is unquestionable. Gross total resection in case of high-grade glioma (HGG) could improve the overall survival of a few months (mean 14 months) at the expense of neurological deficits [1,2]. Although preventing aphasia or hemiplegia is nowadays somewhat obvious in dedicated centers for glioma surgery [3], the damage to the complex language network remains a significant concern for the patient as well as the surgeon in hemisphere-dominant perisylvian tumors [4]. As the surgery needs to preserve the functionality, direct electrical stimulation (DES) in awake patient became the gold standard: performing DES mapping in awake patients with glioma in language eloquent areas results in a significant decrease of postoperative permanent aphasia and more generally of severe persistent neurological deficits [5,6,7].

Besides brain function preservation, DES has contributed to evolve the current model of language pathways. In this model the cerebral language network is organized in two processing streams [8], the dorsal phonological one, involved in auditory-motor association, and the ventral semantic one, supporting the sound-to-meaning mapping [9,10].

Other preoperative techniques have emerged as tools to analyze non-invasively the dual-stream models. Diffusion tensor imaging tractography and language mapping by navigated repetitive transcranial magnetic stimulation (nrTMS) have found their place in clinical practice. The former allows visualization of the language pathway before and during surgery and few works have revealed a relation between fibers injuries during surgical procedures and functional outcome.

Here, a validated method for deterministic tractography was used for preoperative assessment of patients affected by perisylvian glioma [11]. A statistical correlation was investigated between language-relevant fiber bundles and postoperative development of aphasia.

## 2. Materials and Methods

This is a prospective observational study including a series of 18 patients, who underwent surgical resection for a language area associated glioma from August 2019 to August 2020 in the department of Neurosurgery of Turin.

Written and informed consent for all medical evaluation and treatment was obtained from all patients. The following inclusion criteria were used: the presence of a left glioma in proximity to presumed language related brain areas, Italian as a native language and age ≥18 years. The exclusion criteria were general contraindications for MRI, such as metal implants and pregnancy.

The language status was assessed preoperatively, postoperatively (within the first 7 days) and after 3 months. Every patient was examined preoperatively and postoperatively using the Aachen Aphasia Test (AAT) [12], Italian version.

Results of language examination were used to categorize patients into a 4-level aphasia score: 0 = no aphasia (≥90% AAT-score = 0); 1 = mild aphasia (89–75% AAT-score = 1); 2 = moderate aphasia (74–55% AAT = 2); 3 = severe aphasia (<55% AAT-score = 3).

MRI data were acquired on an Ingenia 3T system (Philips Healthcare, Amsterdam, The Netherlands). T1-weighted, T2-weighted, 3D fluid attenuated inversion recovery (FLAIR) and subtraction sequences were performed.

Preoperatively, T1-weighted gadolinium enhanced and T2-weighted/FLAIR (for low-grade tumors) datasets were used to manually segment the tumor and calculate the volume using the Brainlab iPlan software. The extent of tumor resection was assessed by calculating the residual tumor volume using subtraction sequences for high-grade and FLAIR sequences for low-grade gliomas. Gross total resection (GTR) has been considered for volume resection >90%; subtotal tumor resection (STR) has been considered for tumor resection >80% and <90% and partial tumor resection (PTR) have been considered for resection <80%. DTI-based language tractography has been performed using the patient individual, anatomical T1-weighted image as a template.

Five main language tracts were outlined: Arcuate Fascicle (AF), Frontal Aslant Tract (FAT), Inferior Frontal Occipital Fascicle (IFOF), Inferior Longitudinal Fascicle (ILF), Uncinate Fascicle (UF).

Regions of interest (ROIs) were placed at predefined anatomic landmarks within the obligatory pathways of the desired tracts (AF, FAT, IFOF, ILF, UF) according to a standardized protocol previously described [11]. Analysis of MRI images was carried out following the principles of a previous risk stratification model recently proposed [13].

Preoperatively, the distance between tumor and tract was measured by defining the shortest distance of the lesion to the fiber bundle in all three radiological planes. In case of a distance of 0 mm (= tract infiltration), the extent of contact between the tumor and the tract was approximated by calculating the surface intersection of both.

Preoperatively defined tracts were carefully fused with the postoperative images to analyze the potential surgery-related injury of tracts. For a detailed localization of the lesion, each tract was parcellated into three subsegments.

A Pearson’s chi square test was conducted on the data collected. Whether or not a subsegment of each analyzed tract was infiltrated or resected, was related to the postoperative development of persistent aphasia.

The statistical analysis was conducted using an open-source software built on R language (jamovi version 1.6, The jamovi Project (2021); retrieved from https://www.jamovi.org accessed on 21 March 2021). Statistical significance was set at *p* < 0.05.

## 3. Results

Over a period of 1 year, 11 women and seven men with a median age of 52 (24–74) years were included in this study. Surgical procedures were performed for 13 high-grade gliomas, of which seven were located in the frontal lobe and six in the temporal lobe, including one with insular extension and another with involvement of the parietal lobule. Of the five low grade gliomas, four tumors developed in the frontal lobe and one was fronto-parietal.

Gross total resection was achieved in nine patients: seven high grade and two low grade gliomas. Subtotal resection and partial resection have been obtained in six and three patients, respectively. Awake surgery was performed in six cases (three low grade and three high grade lesions) (Table 1).

Preoperative tractography showed an involvement of the outlined tracts in 15 patients out of 18 (83%). In nine out of 18 patients (50%) one or more tracts were damaged resulting in permanent postoperative speech deficit. Only two patients presented a mild transient disorder that regressed to follow up to three months. No patient with pre-operative aphasia showed a significant improvement after surgery (Table 2).

Of the nine patients with permanent speech deficit, four subjects developed mild permanent aphasia (AAT grade 1), three patients developed moderate aphasia (AAT grade 2) and two patients developed severe aphasia (AAT grade 3). In one case the arcuate fascicle was damaged in the temporoparietal segment; in one case AF and FAT were affected; in two cases IFOF ILF AF and for two other AF ILF UF combination were found; FAT in the medial and middle portions has been injured in three patients (Table 3).

The major tract involved in our cohort is frontal-aslant-tract: it was infiltrated in nine cases (50%) and resected in eight cases. This feedback is plausible because most were frontal gliomas. In the four patients with permanent deficits the middle part of FAT has always been damaged.

Arcuate fascicle was the second most involved tract, especially in its temporo-parietal portion. This fascicle was damaged in six patients resulting in permanent speech disorder. In five of them was affected its middle portion (temporo-parietal), in one the frontal segment and in two patients the temporal one was damaged along with the middle portion.

A statistical significance (*p* < 0.05) was reached for resection of middle segment of AF and FAT, which correlate well with the functional outcome. A less strong association has been noted for tracts infiltration. In fact, only the infiltration of the middle portion of AF showed a significant relationship with the postoperative neurological deficit (Table 4).

In the view of the dual stream model, our series shows a greater predisposition to dorsal stream damage. Ventral stream fascicles were involved and resected in fewer cases. Resection of a single portion of these ventral stream tracts did not lead to clinically relevant consequences.

Transitory deficit has been registered in three cases of medial FAT damage, in one patient with lateral FAT segment injury and in one case of frontal IFOF injury. In the remaining four patients without post-operative speech disorder, these language fascicles were not resected.

## 4. Discussion

**Figure 1:**
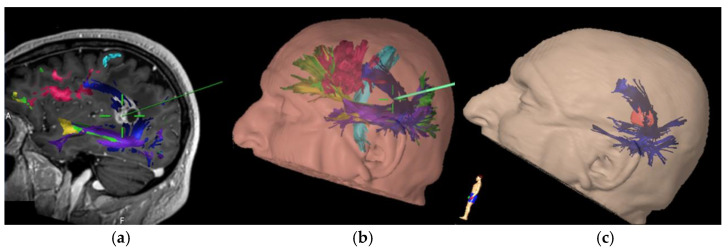
Intraoperative screenshots with navigated tractography of left temporo-parietal high-grade glioma involving the middle part of AF (**a**) T1 contrast enhanced MRI; (**b**) 3d volume rendering with coloured tracts; (**c**) 3d volume rendering with AF and tumor.

This study shows that standardized reconstruction based on DTI sequences of language fascicles can effectively predict the risk of developing postoperative speech disorder in treatment of perisylvian brain tumors. In all nine patients who developed a permanent disorder there was the pre-operative involvement of at least one fiber bundle and injury of at least one of these tracts. The larger volume of pre-operative infiltration was estimated, the greater incidence of a new aphasic disorder was observed. In the five patients with impaired tracts without permanent damage, the resection volume has been less than 0.5 cm^3^. Four patients developed mild permanent aphasia (AAT 1) and for three of them the middle FAT had been resected. The main problem in these subjects was the difficulty to begin to speak, some presented stuttering, and language was not normally fluent. In the fourth case of mild disturbance the combination of resection of AF ILF UF was observed. Three patients had moderate aphasia (AAT 2) with resection or AF alone or in combination with FAT. In two cases severe aphasia (AAT 3) was found and both dorsal and ventral stream were compromised (AF ILF IFOF and AF ILF UF). In the contemporary model of language organization, it is believed that the arcuate fascicle plays an essential role of connection between frontal and temporal areas constituting the main component of the dorsal stream. According to our findings, preoperative infiltration as well as surgical lesions of AF turned out to be an individual risk factors for deterioration of linguistic function. The temporo-parietal subsegment of the AF has shown to have the strongest association with the development of permanent aphasic disorder when involved during surgical resection. This suggests a greater vulnerability of the dorsal linguistic network in case of AF infiltration (Figure 1), possibly due the lack of compensation mechanisms. Conversely, compensation mechanisms have been described for ventral stream in which accessory pathways via ILF and UF can be used in permanent IFOF lesions [14].

**Figure 2:**
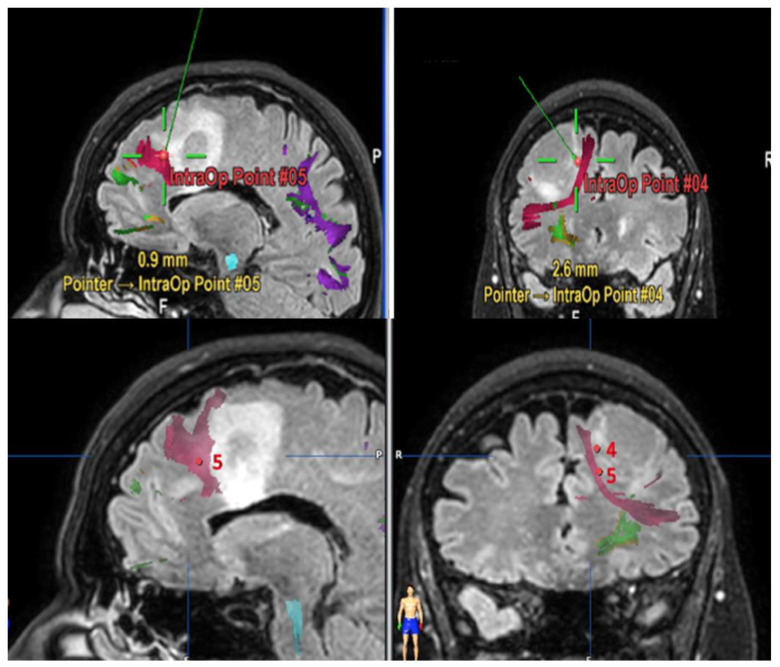
Intraoperative screenshots of FLAIR MRI sequences with navigated tractography of left frontal low-grade glioma. Point 4 corresponds to the stimulation of the medial part and the point 5 to the middle part of FAT.

**Figure 3:**
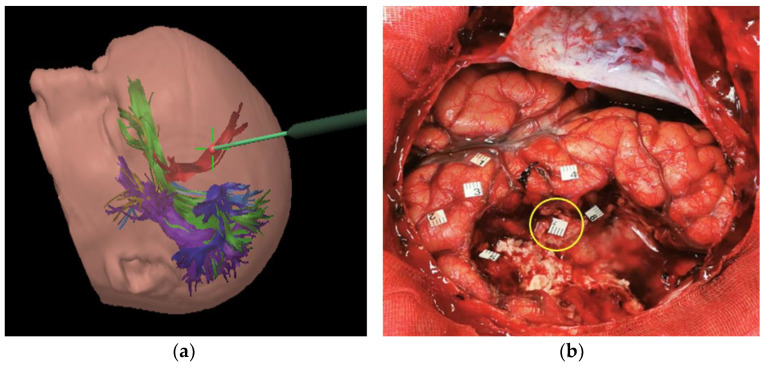
(**a**) Intraoperative 3D image showing the pointer of neuronavigation system positioned at the level of the surgical cavity that encounters the FAT (red fascicle); (**b**) Intra-operative picture of the surgical cavity during resection of left frontal low-grade glioma indicating the stimulated point (n. 7) at the middle part of FAT causing speech arrest.

A separate consideration deserves FAT. Some reports [15] suggest that lesions of this fascicle are not associated with postoperative permanent speech disorder. On the contrary, our results show that this tract plays a significant role in verbal fluency and proper motor initiative of speech. In seven out of 12 subjects where this tract was involved during surgical resection, a permanent aphasic disorder, mainly regarding verbal fluency, was observed. In particular, all the patients that underwent surgical resection for lesions involving the middle section (from the upper frontal sulcus to the middle frontal sulcus) of the FAT (Figure 2), presented postoperatively a permanent deficit in verbal initiative. This correlation is strongly supported by intraoperative experience in the three cases carried out in awake surgery, in which direct subcortical navigated stimulation of the tract immediately caused speech arrest (Figure 3). In these cases, surgical resection was interrupted, avoiding postoperative permanent damage. In the remaining patients who underwent asleep surgery, and a portion of the FAT was injured, there was a verbal fluency disorder that also persisted in follow-up. Specifically, four cases were found for middle FAT and three cases for medial FAT, while none for the lateral part of the tract.

The ventral stream of language consists of areas of occipital, temporal and frontal cortex connected by white matter fascicles mainly represented by IFOF, ILF and UF. These tracts are involved in receiving sensory, visual or auditory inputs and processing semantic information to facilitate language comprehension [8].

**Figure 4:**
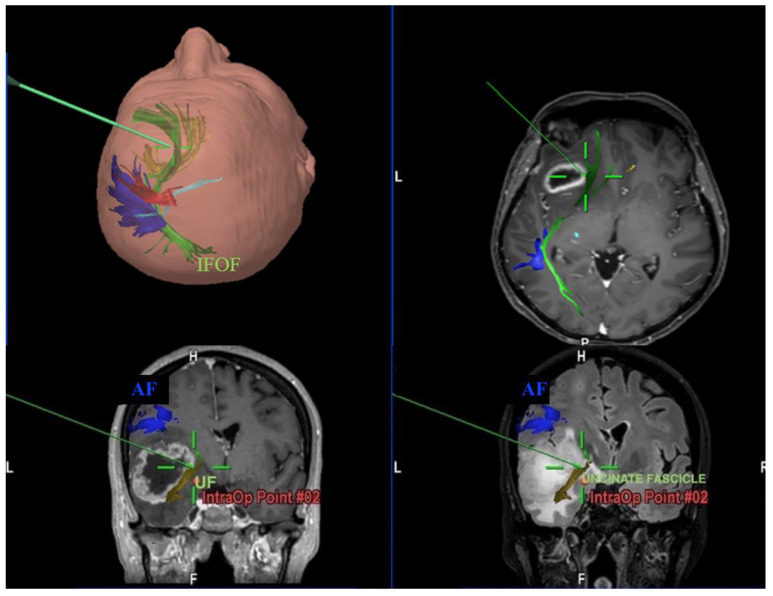
Intraoperative screenshots with navigated tractography of left high-grade gliomas showing the pointer of neuronavigation system that encounters IFOF (above) and UF (below).

According to our results, none out of the eighteen patients presented a permanent disorder when a single ventral stream fascicle was involved during surgery. Post-operative language disorders emerged as result of simultaneous fascicles injury (IFOF ILF and ILF UF for two patients each) (Figure 4). This could suggest the existence of a crucial white matter area where lesions in the temporal stem, in which IFOF, middle ILF and middle UF converge, could lead to a disconnection of the direct and indirect pathways in the ventral steam and therefore less possibility of post-lesional reorganization according to mechanisms of neuronal plasticity. It can thus be stated, based on an objective volumetric evaluation, that lesions of the intermediate part of a fiber bundle, where most axonal fibers converge, likely leads to a wide disconnection of the cortical areas involved in a functional network compared to the lesion on the cortical ends of a tract. Since previous experience and direct observation of these patients in the post-operative period, it is likely to believe that the adult brain hides incredible resilience after suffering an injury [16]. However, these potentials in some cases seem to be limited, especially when certain parts of white matter are damaged [17].

Our results seem to agree with previous studies in which it has been suggested the existence of functional nodes within functional networks so that once they become damaged, even small portions, they lose that ability to recover. A rigorous data selection was used in which patients were followed with a reproducible test and tractographies were analyzed with an already validated method [13]. Data have been obtained on the importance of some subcortical areas which are often affected by gliomas. These subcortical hubs for linguistic function can be intended by the temporoparietal junction containing the temporoparietal segment of AF and the middle frontal white matter where the mid-segment of the FAT is located for the dorsal stream [18]. The temporal stem, containing the central segment of the IFOF and the ILF and the UF, is the core of the ventral stream [19]. The middle part of the arcuate fascicle and the FAT and the temporal stem appear to be the most relevant in the processes of language and therefore the most delicate when approaching with the resection of an intra-axial lesion. Navigated tractography allows a likely non-invasive and in vivo reconstruction of the white matter fascicles during surgical procedure. This technique has become increasingly widespread in planning and execution of neurosurgical procedures, mostly in glioma surgery [20].

Various studies have shown the value of the tractography for perioperative risk assessment and surgical planning [21,22,23]. Despite the evidence in the clinical use, others criticize the method for its technical characteristics and the possible misinterpretation of the results [24]. With the introduction of a standardized tractography protocol it is considered to overcome the known shortcomings of the technique to allow the better use in neurosurgical clinical practice [11]. After dealing with the planning of these 18 patients, we are confident that the intraoperative tractography can quickly to be part of every modern neurosurgeon’s armamentarium. Given the results of this study, we propose the power of this standardized tractographic approach in surgical planning and risk stratification in patients with gliomas in speech eloquent areas. It is known that these tumors can significantly distort white matter fascicles and displace them in unexpected areas. Low-grade tumors tend mainly to infiltrate these bundles while high grade gliomas, producing edema, tend to reduce axonal density and displace them more frequently. Tractography fiber bundles can underestimate the real volume of the fascicles, and this is a limit of the technique. However, there were no insurmountable technical difficulties, so all 5 language tracts were outlined for each patient. The tractography work was carried out exclusively by the neurosurgeons and it is emphasized that the involvement of the neurosurgeon is paramount to obtain good quality data.

A correlation has been found between pre-operative infiltration, tracts resection, and post-operative speech deficit. Therefore, it can be considered that navigated tractography is a user friendly, non-invasive, and relatively fast tool both for image acquisition times (less than 20 min) and for planning processing (less than 60 min per patient in our cases). It is used to assess the specific operation risk in each patient. This technique emphasizes the relevance of subcortical connectivity in the modern view of oncological neurosurgery. Damaging relevant functional nodes leads to loss of potential pathways for neural plasticity. The navigated tractography helps neurosurgeon to find a balance between tumor resection and neurological outcome allowing to delineate more easily that labile boundary between tumor and brain parenchyma. Information obtained can be used to tailor the best surgical trajectory individually and judge the level of aggressiveness of a lesion when it has a high degree of dislocation or infiltration of the outlined fascicles. It is now possible to predict with a certain reliability the risk of having permanent damage following surgery. In addition, the tractographic guide can allow to perform targeted subcortical stimulations and reduce the time of surgery during awake procedures. While recognizing the limits of the method, which is a probable in vivo representation of white matter tracts, the relevant role of the same is recognized in helping neurosurgeon in the difficult task of maximizing the extent of resection preserving neurocognitive outcome. This method is not in contrast to other techniques to increase the accuracy of resection but implements the possibilities of the much sought-after maximal safe resection alongside the use of direct stimulation to awake patients and fluorescence guided surgery [25,26]. To increase the extent of resection it is possible to combine the use of 5-ALA fluorescence with contrast-enhanced ultrasound [27]. This strategy could help to visualize more tumor tissue than one tool alone. However, it is crucial to preserve functional areas highlighted by navigated in order to ensure the onco-functional balance.

Intraoperative navigated tractography seems to be a viable alternative to guide resection in all those patients not eligible for awake surgery. In cases where awake surgery is proposed, this technique guides the subcortical stimulation during the procedure leading to a more efficient identification of eloquent pathways and a decrease in surgical time [28,29]. Double iconographic and electrophysiological confirmation during awake procedures may allow the neurosurgeon to weigh the choice to be conservative or aggressive in the resection. The number of patients in this study limits the possibility to assess the exact risks associated with the involvement and the damage of the analyzed fascicles. Therefore, future studies are expected to increase the number of patients operated and evaluated according to objective and reproducible criteria to obtain more significant results. Results of this study depend solely on the radiological results that have been correlated to the state of the postoperative speech ability. Despite efforts to avoid misalignments when merging preoperative and post-operative images using distortion correction tool and individual manual correction, errors due to distortion for the “brainshift” or scanning procedures cannot be excluded. Limits of deterministic tractography are known: it is not able to recognize the difference between afferent and efferent connections and the crossing fibers remain of difficult interpretation [30].

Further consideration must be given to the fact that the post-operative deficit may have been caused by direct cortical injury or combined with subcortical damage. A detailed speech evaluation carried out with the AAT test can highlight a higher incidence of aphasic disorder that in everyday life can be little appreciated. It would also deserve an assessment of the impact on the quality of life of the disorders detected, as well as the use of other language tests in order to detail more precisely the post-operative aphasic disorders.

## 5. Conclusions

Glioma surgery is definitely complex. Management of these patients must be multidisciplinary, and surgery plays a fundamental role, representing the first step of treatment on which the following depend. It is therefore crucial to use all the tools that modern medicine offers in order to remove as much as possible tumor while preserving quality of life of the patient.

The present study seems to support the hypothesis that results obtained from the pre-operative tractography, navigated during the surgical procedure, well correlate with the functional outcome of patients affected by gliomas undergoing surgery in so-called speech eloquent areas.

This method optimizes the evaluation of preoperative risk, guides intraoperative resection, correlates with postoperative deficits, and can reduce the time of awake surgery. A greater use of direct intraoperative stimulation in awake surgery can be valuable to prove the reliability of the method. The aim is to further validate the routinary use of navigated tractography for speech eloquent areas glioma surgery and other pathologies. Our results underline the importance of customizing surgery to minimize functional damage to the benefit of the patients.

## Figures and Tables

**Table 1:** Summary of tumor location, extent of resection and surgery; GTR, gross total resection, STR, subtotal resection; PR, partial resection; LGG, low grade glioma; HGG high grade glioma.

	LGG	HGG	Total
No. of patients	5	13	18
**Tumor location**			
Frontal	4	6	10
Temporal	0	5	5
Fronto-parietal	1	1	2
Temporo-insular	0	1	1
**Extent of resection**			
GTR	2	7	9
STR	1	5	6
PR	2	1	3
**Surgery**			
Awake	3	3	6
Asleep	2	10	12

**Table 2:** Infiltered and injured tracts.

Tracts	Infiltration (*n* = 15)	Injured Tracts with Permanent Aphasia (*n* = 9)	Injured Tracts without Aphasia (*n* = 5)
Frontal AF	3	1	0
Temporo-parietal AF	5	5	0
Temporal AF	3	2	0
Medial FAT	7	3	3
Middle FAT	4	4	0
Lateral FAT	1	0	1
Frontal IFOF	2	0	1
Middle IFOF	3	2	0
Temporal ILF	2	2	0
Middle ILF	2	2	0
Temporal UF	3	2	0

**Table 3:** Grade of postoperative speech deficits.

Aphasia Grade	Temporo-Parietal AF (11%)	AF-FAT (11%)	Middle ± Medial FAT (34%)	AF-ILF-UF (22%)	AF-ILF-IFOF (22%)
AAT 1	0	0	3	1	0
AAT 2	1	1	0	0	1
AAT 3	0	0	0	1	1

**Table 4:** Statistical analysis for postoperative persistent aphasia in perisylvian left-hemisphere glioma. N is the number of non-missing value. 1, Kruskal-Wallis. 2, Pearson.

		N	0	1	Test Statistic		N	0	1	Test Statistic
			(N = 9)	(N = 9)				(N = 9)	(N = 9)	
	Infiltration: 1					Resection: 1				
AF	frontal	18	0.1 1/9	0.1 1/9	Χ$\begin{array}{c} 2\\ 1 \end{array}$ = 0.00, *p =* 1.00^2^	frontal	18	0.1 1/9	0.1 1/9	Χ$\begin{array}{c} 2\\ 1 \end{array}$ = 0.00, *p =* 1.00^2^
	middle	18	0.0 0/9	0.4 4/9	Χ$\begin{array}{c} 2\\ 1 \end{array}$ = 5.14, *p =* 0.02^2^	middle	18	0.0 0/9	0.6 5/9	Χ$\begin{array}{c} 2\\ 1 \end{array}$ = 6.92, *p =* 0.01^2^
	temporal	18	0.0 0/9	0.3 3/9	Χ$\begin{array}{c} 2\\ 1 \end{array}$ = 3.60, *p =* 0.06^2^	temporal	18	0.0 0/9	0.2 2/9	Χ$\begin{array}{c} 2\\ 1 \end{array}$ = 2.25, *p =* 0.13^2^
ILF	temporal	18	0.0 0/9	0.2 2/9	Χ$\begin{array}{c} 2\\ 1 \end{array}$ = 2.25, *p =* 0.13^2^	temporal	18	0.0 0/9	0.3 3/9	Χ$\begin{array}{c} 2\\ 1 \end{array}$ = 3.60, *p =* 0.06^2^
	middle	18	0.0 0/9	0.2 2/9	Χ$\begin{array}{c} 2\\ 1 \end{array}$ = 2.25, *p =* 0.13^2^	middle	18	0.0 0/9	0.2 2/9	Χ$\begin{array}{c} 2\\ 1 \end{array}$ = 2.25, *p =* 0.13^2^
IFOF	temporal	18	0.2 2/9	0.1 1/9	Χ$\begin{array}{c} 2\\ 1 \end{array}$ = 0.40, *p =* 0.53^2^	temporal	18	0.1 1/9	0.1 1/9	Χ$\begin{array}{c} 2\\ 1 \end{array}$ = 0.00, *p =* 1.00^2^
	middle	18	0.0 0/9	0.3 3/9	Χ$\begin{array}{c} 2\\ 1 \end{array}$ = 3.60, *p =* 0.06^2^	middle	18	0.0 0/9	0.2 2/9	Χ$\begin{array}{c} 2\\ 1 \end{array}$ = 2.25, *p =* 0.13^2^
UF	frontal	18	1.0 9/9	1.0 9/9	NA	frontal	18	1.0 9/9	1.0 9/9	NA
	middle	18	0.0 0/9	0.1 1/9	Χ$\begin{array}{c} 2\\ 1 \end{array}$ = 1.06, *p =* 0.30^2^	middle	18	1.0 9/9	1.0 9/9	NA
	temporal	18	0.0 0/9	0.2 2/9	Χ$\begin{array}{c} 2\\ 1 \end{array}$ = 2.25, *p =* 0.13^2^	temporal	18	0.0 0/9	0.2 2/9	Χ$\begin{array}{c} 2\\ 1 \end{array}$ = 2.25, *p =* 0.13^2^
FAT	frontosuperior	18	0.4 4/9	0.3 3/9	Χ$\begin{array}{c} 2\\ 1 \end{array}$ = 0.23, *p =* 0.63^2^	frontosuperior	18	0.3 3/9	0.3 3/9	Χ$\begin{array}{c} 2\\ 1 \end{array}$ = 0.00, *p =* 1.00^2^
	middle	18	0.1 1/9	0.4 4/9	Χ$\begin{array}{c} 2\\ 1 \end{array}$ = 2.49, *p =* 0.11^2^	middle	18	0.0 0/9	0.4 4/9	Χ$\begin{array}{c} 2\\ 1 \end{array}$ = 5.14, *p =* 0.02^2^
	frontoinferior	18	0.1 1/9	0.0 0/9	Χ$\begin{array}{c} 2\\ 1 \end{array}$ = 1.06, *p =* 0.30^2^	frontoinferior	18	0.1 1/9	0.0 0/9	Χ$\begin{array}{c} 2\\ 1 \end{array}$ = 1.06, *p =* 0.30^2^
